# Design of a Functional Pea Protein Matrix for Fermented Plant-Based Cheese

**DOI:** 10.3390/foods11020178

**Published:** 2022-01-11

**Authors:** Carmen Masiá, Poul Erik Jensen, Iben Lykke Petersen, Patrizia Buldo

**Affiliations:** 1 Department of Food Science, University of Copenhagen, Rolighedsvej 26, 1958 Frederiksberg, Denmark; peje@food.ku.dk (P.E.J.); ilp@food.ku.dk (I.L.P.); 2 Plant Based Application Department, Chr. Hansen A/S, Bøge Alle 10-12, 2970 Hørsholm, Denmark; patrizia.buldo@gmail.com

**Keywords:** plant-based cheese, pea protein, gel, emulsion, stability, functionality

## Abstract

The production of a fermented plant-based cheese requires understanding the behavior of the selected raw material prior to fermentation. Raw material processing affects physicochemical properties of plant protein ingredients, and it determines their ability to form fermentation-induced protein gels. Moreover, the addition of oil also influences structure formation and therefore affects gel firmness. This study focuses on identifying and characterizing an optimal pea protein matrix suitable for fermentation-induced plant-based cheese. Stability and gel formation were investigated in pea protein matrices. Pea protein isolate (PPI) emulsions with 10% protein and 0, 5, 10, 15, and 20% olive oil levels were produced and further fermented with a starter culture suitable for plant matrices. Emulsion stability was evaluated through particle size, ζ-potential, and back-scattered light changes over 7 h. Gel hardness and oscillation measurements of the fermented gels were taken after 1 and 7 days of storage under refrigeration. The water-holding capacity of the gels was measured after 7 days of storage and their microstructure was visualized with confocal microscopy. Results indicate that all PPI emulsions were physically stable after 7 h. Indeed, ζ-potential did not change significantly over time in PPI emulsions, a bimodal particle size distribution was observed in all samples, and no significant variation was observed after 7 h in any of the samples. Fermentation time oscillated between 5.5 and 7 h in all samples. Higher oil content led to weaker gels and lower elastic modulus and no significant changes in gel hardness were observed over 7 days of storage under refrigeration in closed containers. Water-holding capacity increased in samples with higher olive oil content. Based on our results, an optimal pea protein matrix for fermentation-induced pea protein gels can be produced with 10% protein content and 10% olive oil levels without compromising gel hardness.

## 1. Introduction

Limited knowledge about plant protein behavior during and after processing the protein ingredient leads to low-quality plant-based products. When it comes to cheese alternatives, their production requires reverse engineering: Taking a hard gel as the final aim and designing a matrix with gelling properties to mimic it. Commercial plant-based drinks generally contain limited amounts of proteins and therefore, they are not suitable matrices for protein-rich gels. Fermented plant-based cheese production requires a specific protein-rich matrix with the ability to form a protein gel upon fermentation. Moreover, it should be a liquid matrix to enable homogeneous bacterial inoculation. Since fat is an essential component in cheese, and plays a major role in mouth feel, oil-in-water emulsions rather than protein suspensions without oil are desired for plant-based cheese matrices. The design of this matrix should consider two main aspects: Stability and gel formation ability.

The liquid matrix must remain stable and homogeneous during fermentation, and only show changes due to the effect of microorganisms, fermentation conditions, or applied treatments to modify its texture. Fermentation time is microorganism-dependent, and phase separation must be avoided over the course of fermentation. One of the factors affecting stability is protein solubility, which is influenced by the interactions proteins establish with the solvent and with other proteins. Proper hydration of charged amino acids encourages protein-solvent interactions [1], and therefore hinders phase separation. Other ways of improving protein solubility are increasing protein steric repulsion, increasing protein electrostatic repulsion (adjusting pH away from their isoelectric point), or reducing protein hydrophobic attractions, among others [2]. Another factor influencing matrix stability is oil droplet size, a critical parameter to avoid oil-droplet aggregation and creaming during fermentation and shelf life [3].

Functionality is application-dependent. For instance, in plant-based drinks, the desired protein functionality is solubility. However, in fermentation-induced plant-based cheese, the desired protein functionality is gelling ability, as proteins must turn into a three-dimensional gel network during fermentation [4]. Two aspects of plant protein functionality have recently generated interest. One aspect is the status of the proteins, which is affected by raw material processing. Treatments involving high temperatures or drastic pH changes have an impact on matrix components on a molecular level while potentially affecting their gelation capacity. Purification at high temperatures or heat sterilization partially or totally unfolds proteins, exposing their hydrophobic residues. This encourages protein-protein interaction and leads to aggregation and protein network formation. Even though plant seed proteins reach their highest solubility at its native structure [5], a certain degree of denaturation is needed for gel formation. Moreover, gelation is dependent on the protein denaturation level [6]. Another aspect is the purity of protein fractions. Most of the work done in the past decades has focused on protein isolates (80–95% protein) to assess pea protein functionality. However, recent studies suggest that high degree of purification is unnecessary to benefit from pea protein functionality [7] and that less-processed ingredients yield stronger heat-induced gel structures [8]. Nevertheless, in fermented pea protein gels, pasteurizing the matrix before fermentation is necessary to ensure safety and unique growth of the inoculated starter culture. If the matrix contains high starch content, from a non-hydrolyzed concentrate or flour, it will gelify prior to fermentation and it will not be possible to inoculate bacteria in a liquid media. Therefore, less processed ingredients can be of interest in other applications, but for fermentation-induced pea protein gels, they can present challenges during matrix processing.

Yellow peas have become one of the most acclaimed raw materials for the development of plant-based alternatives due to their high protein content, availability, and low cost production [9]. Pea globulins (70–80% of the total protein content [9], constituted mainly by legumins and vicilins) play the main role in gel structure formation [10], which follows three stages: (1) Denaturation of protein and exposure of hydrophobic residues, (2) intermolecular hydrophobic interaction of unfolded proteins, and (3) agglomeration of aggregates into a three-dimensional network that englobes water, fat, and other matrix components [11]. Numerous studies have already investigated gelation mechanisms of pea protein. Most of them focus on heat-induced pea protein gels [8,12,13], but others also explore high pressure-treated gels [14] or cold-set gels through acidification with glucono-δ-lactone [4,15]. However, only a few explore fermentation-induced pea gels. Yousseef et al. worked with a mixture of pea proteins and milk proteins [16], and Verma et al. and Ben-Harb et al. fermented a pea protein matrix but did not assess the texture of their fermented product [17,18]. Despite vicilin or legumin playing the main role in gel formation, Klost et al. reported the importance of the type of interactions over the protein fraction forming the gel structure [19]. These interactions are influenced by the protein structure, which is directly affected by raw material processing [13]. Most of the studies on pea-based gels target plant-based applications such as yogurt [20,21], meat, and seafood analogues [6], but few focus on cheese alternatives [4,17]. Moreover, the majority of the currently commercialized plant-based cheeses (fermented or not) rely on crystallized fats such as coconut oil to develop firm textures. Others do not include texturing agents in their formulations but their consistency is not hard enough for a sliceable product. Pea proteins have a big potential for gel formation. However, they require structure unfolding to interact with each other and form three-dimensional structures [20] with the hardness of a firm gel.

This study focuses on the stability and gel formation capacity of a pea protein matrix for fermentation-induced protein gels. Both parameters are directly affected by matrix composition and the processing it undergoes. Stability was evaluated through particle size and ζ-potential changes over time, combined with emulsion stability assessment performed with back-scattered light detector. Functionality was evaluated by assessing gel formation through texture analysis, rheological measurements, water-holding capacity measurements, and confocal microscopy visualization. This study is relevant for the research community as well as for the industry players that are exploring the world of fermented plant-based cheese and facing texture-related challenges.

## 2. Materials and Methods

### 2.1. Materials

The pea protein isolate (PPI) used in this study was ProFam^®^580 (ADM, Chicago, IL, USA). The declared composition is: 81.3% protein, 7% fat, and 9% fiber. Other matrix ingredients were extra-virgin olive oil produced in Spain (Salling Group, Ishøj, Denmark), sucrose, and glucose (Sigma Aldrich, Søborg, Denmark). The starter culture used for fermentation was Vega^TM^ Harmony (Chr. Hansen A/S, Hørsholm, Denmark). The strains present in this starter culture are *Streptococcus thermophilus* and *Lactobacillus bulgaricus* supplemented with *Lactobacillus acidophilus*, *Lactobacillus paracasei*, and *Bifidobacterium*.

### 2.2. Methods

#### 2.2.1. Preparation of Pea Matrices

**Figure 1:**
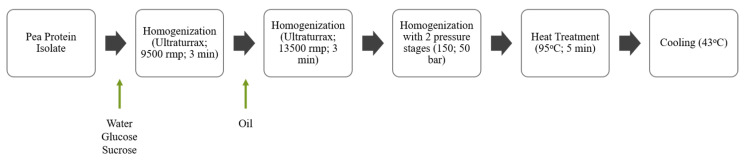
Production process of a pea protein isolate (PPI) matrix for plant-based cheese.

PPI was suspended in distilled water to a final protein concentration of 10%. To ensure protein hydration, the mixture was stirred at 9500 rpm with a T 25 digital ultra-turrax^®^ (IKA, Staufen, Germany) for 3 min. Glucose and sucrose were added in this first stirring step at a concentration of 1% each to ensure that there is substrate for the bacteria to grow and acidify the matrix, given the low sugar content of the pea protein isolate used in this study. Samples were emulsified with different olive oil concentrations, namely 0, 5, 10, 15, and 20%, and labeled as E0, E5, E10, E15, and E20, respectively, with ultra-turrax at 13,500 rpm for 3 min and were subjected to homogenization in an GEA Lab Homogenizer PandaPLUS 2000 (GEA, Parma, Italy) at two stages (150 and 50 bars) in one pass. Homogenized emulsions were pasteurized at 95 °C for 5 min in a water bath and cooled down to 43 °C prior to microbial inoculation (0.02% inoculum). Bottles and containers used for matrix preparation were previously dry autoclaved at 121 °C for 20 min. Flow diagram of the matrix production process can be seen in Figure 1.

For stability measurements, two separate batches of each PPI liquid matrix were produced for each experiment and three technical replicates were measured for each. For gel formation assessment, three separate batches of each fermented gel were produced for each experiment. All hardness and rheology measurements were performed in biological triplicates with three technical replicates each.

#### 2.2.2. Physical Stability

Emulsion stability was determined with Turbiscan^LAB^ (Formulaction, Toulouse, France). Cylindrical glass tubes with 5 mL of each emulsion were introduced in the reader. The system was programmed to measure the intensity of transmitted and back-scattered light over the whole tube height at t = 0 h and t = 7 h. Samples were stored at room temperature between measurements. The percentage of back-scattered light at 40 mm in the tube at t = 0 h was compared to that at t = 7 h.

#### 2.2.3. Particle Size and ζ-Potential Measurements

Particle size and ζ-potential of pea protein emulsions were measured using laser diffraction and dynamic light scattering with Mastersizer 3000 and Zetasizer Nano-ZS 900, respectively (Malvern Panalitical, Malvern, United Kingdom). For particle size measurements, samples were diluted in the degassed water tank coupled to the equipment until reaching obscuration ratios between 12% and 20%. For ζ-potential measurements, mixtures were diluted 100 times in deionized water and loaded in a special capillary cuvette with two electrodes. All measurements were performed in triplicate with freshly prepared samples at a constant temperature of 20 °C and at an angle of 90°, using a refractive index (RI) of 1.52 for pea protein’s ζ-potential [22] and 1.47 for olive oil in particle size measurement [23]. Particle size in the sample without oil was also measured with olive oil’s RI as a background control. Measurements were taken at t = 0 h. Samples were stored at room temperature and measurements were repeated at t = 7 h.

#### 2.2.4. Fermentation of Pea Matrices

Pasteurized emulsions with different oil concentrations were fermented with 0.02% bacterial inoculum of Vega^TM^ Harmony (Chr. Hansen A/S, Hørsholm, Denmark) in a water bath at 43 °C. pH was measured every 5 min with iCinac (AMS S.R.L., KPM Analytics, Rome, Italy). When pH 4.5 was reached, fermented samples were stored under refrigeration at 5 °C. Fermented samples were named according to their oil content, namely G0, G5, G10, G15, and G20.

#### 2.2.5. Textural Characterization

##### Texture Analysis

A puncture test was performed with a Texture Analyzer (Stable Micro Systems, Surrey, United Kingdom) to evaluate the maximum force required to penetrate the fermented samples. A cylindrical probe (TA-510 10-mm diameter, 45-mm long, stainless steel) penetrated the sample to a depth of 6 mm at a 1 mm/s speed, and returned at 10 mm/s speed. Hardness was calculated by Exponent 32 software (Stable Micro Systems, Surrey, United Kingdom) as the maximum peak value detected during sample compression. Texture analysis was performed after 1 day and 7 days of storage under refrigeration at 5 °C in closed containers.

##### Rheological Measurements

A preliminary amplitude sweep test from 0.01% to 100% strain at 1 Hz was performed on each sample in a Kinexus rheometer (Netzsch-Gerätebau GmbH, Selb, Germany) to determine the linear viscoelastic region (LVR). After analyzing the obtained results (data not shown), 0.1% strain was selected to perform a frequency sweep test from 0.01 Hz to 10 Hz on each gel. These tests were performed with a parallel plate-to-plate geometry with flat surfaces (20-mm diameter top plate and 65-mm diameter low plate) and a 1-mm gap. Rheological measurements were taken after 1 day and 7 days of storage under refrigeration at 5 °C in closed containers. For the technical replicates, three different slices of each biological replicate were used.

#### 2.2.6. Gel Structure Visualization

Fermented gels after 7 days of storage under refrigeration in closed containers were visualized at room temperature at a microstructural level with a Leica SP5 (D102) confocal laser scan microscope (CLSM) (Leica Microsystems GmbH, Wetzlar, Germany). Samples were previously stained with fluorescent dyes. A total of 10% (*v*/*v*) Nile blue (10 mg/mL) dissolved in water was used to visualize protein and 1% (*v*/*v*) Nile red (0.5 mg/mL) dissolved in acetone was used to visualize oil droplets. Excitation wavelengths of 633 nm and 488 nm were used for protein and oil droplets observation, respectively. A 63x oil immersion objective was used to observe the stained samples.

#### 2.2.7. Water-Holding Capacity

Water-holding capacity (WHC) of fermented samples was evaluated after 7 days of storage under refrigeration at 5 °C following a slightly modified Sharma et al. method [24]. A total of 5 g of each gel were centrifuged at 6500× *g* at 10 °C for 20 min. WHC (%) was defined according to Equation (1),

(1) $$WHC\left(\%\right)=\left(Water_{total}-Water_{expelled}\right)*100$$

where *Water_total_* is the total water content in the sample (g) and *Water_expelled_* is the expelled water after centrifugation (g).

#### 2.2.8. Statistical Analysis

The effect of time on ζ-potential and particle size was analyzed with unpaired *t*-tests. Physical stability measurements were analyzed with paired *t*-tests. The effect of time on hardness and rheological measurements was evaluated with unpaired *t*-tests, and significant differences between PPI gels were identified through ANOVA followed by Tukey test. Significant differences were assumed when *p* < 0.05.

## 3. Results and Discussion

### 3.1. Matrix Formulation

Preliminary tests concluded that 10% protein is an optimal protein concentration for a stable liquid matrix prepared with the PPI used in this study (data not shown). This concentration kept the density of the matrix low, allowing easy pre-processing and homogeneous inoculation in a liquid media. Moreover, Munialo et al. [25] reported no significant differences in pea gel microstructures at a fixed pH and different protein concentrations. However, they observed significant alterations at different pH ranges and constant protein concentration [25]. This supports the use of fermentation to induce pea protein reorganization into a different structural network by keeping the protein content constant. Olive oil was included at different concentrations (0, 5, 10, 15, and 20%) to provide the matrix with the fat that is needed for a cheese-like product and to stabilize pea proteins in suspension, which will likely sediment over time if only suspended in water. The initial pH of PPI emulsions was 7.

### 3.2. Physical Stability of PPI Emulsions

**Figure 2:**
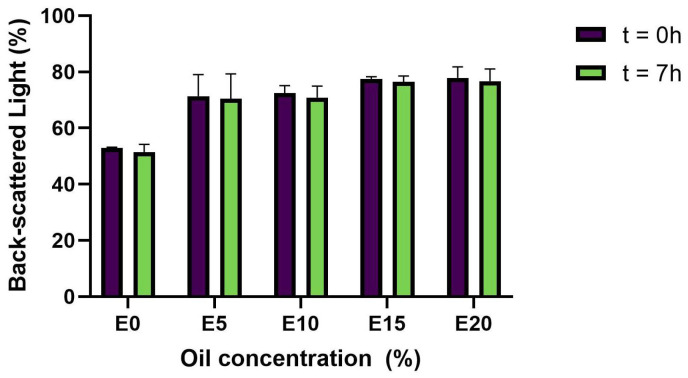
Back-scattered light in PPI emulsions at t = 0 h and t = 7 h under storage at room temperature. Sample name corresponds to its oil content, e.g., E0: 0% oil, E5: 5% oil, etc.

Physical stability is the ability of a system to avoid physical changes over time [26]. Emulsions are unstable systems due to the immiscibility of water and oil, and they tend to present phase separation over time. Therefore, the use of surfactants as emulsifier agents prevents oil droplet aggregation [27]. In the system produced in this study, pea protein acted as a surfactant due to its great emulsifying properties [1,28] and contributed to emulsion stability. Physical stability was evaluated by comparing back-scattered light percentages in a specific point of the tubes containing the samples at two different time points. The percentage of back-scattered light in each PPI matrix did not change significantly after 7 h of storage at room temperature (Figure 2). These results confirm that, independent of the amount of oil, all matrices were physically stable for at least 7 h and suitable for fermentation.

### 3.3. Oil Droplet Aggregation

**Figure 3:**
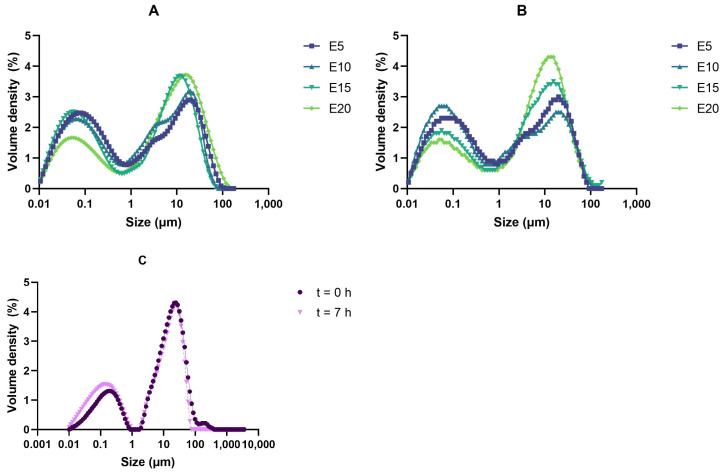
Volume density of particle size in PPI emulsions at (**A**) t = 0 h, (**B**) t = 7 h, and (**C**) PPI suspensions without oil (E0) after storage at room temperature. Sample name corresponds to its oil content, e.g., E0: 0% oil, E5: 5% oil, etc.

Homogenization with 2 pressure stages (50 and 150 bar) was applied to all samples to decrease oil droplet size and avoid phase separation. Bi-modal particle size distributions were observed in all samples (Figure 3), as previously reported in plant drinks studies [29]. To evaluate particle size changes over time, the highest points of the two identified peaks at t = 0 h were compared to those at t = 7 h. None of the peak values changed significantly over time in any of the samples, reflecting emulsion stability. Increasing oil content revealed an increase in the amount of the larger oil droplets. The protein in the matrices was probably not enough to cover the entire oil droplet surface and therefore, small oil droplets coalesced. When using the RI of olive oil, particles were detected in PPI suspensions where no oil was included (Figure 3C). This could be explained by the fact that the RI of pea protein (1.52) is very close to that of olive oil (1.47). Therefore, it it not possible to identify pea protein or oil droplets separately but particles composed by both of them.

### 3.4. Electrostatic Interactions between Proteins

Electrostatic interactions between pea proteins are the main factor in protein aggregation [30] and are represented by the ζ-potential. Values in all matrices (pH 7) oscillated between 30 mV and 35.8 mV (absolute values) (Table 1). Piorkowski et al. reported ideal absolute values of at least 20 mV for long-term stability of beverage emulsions [31], which confirms the high stability of our matrices, achieved with weak electrostatic interactions and high net charge. ζ-potential did not change significantly over time in any of the PPI matrices containing oil. This suggests that protein surface charges remained stable and electrostatic repulsion between them was enough to avoid their interaction. Proteins can adsorb to the surface of the oil droplets and, besides not forming protein aggregates, they can hinder oil droplet coalescence and increase emulsion stability [1]. However, ζ-potential of PPI suspensions without oil significantly increased over time, although the difference was not large. A reasonable explanation for this outcome could be that proteins were slowly hydrated over time, unfolding in contact with water, exposing charges, and creating greater repulsion between themselves.

### 3.5. Gel Formation during Fermentation

**Figure 4:**
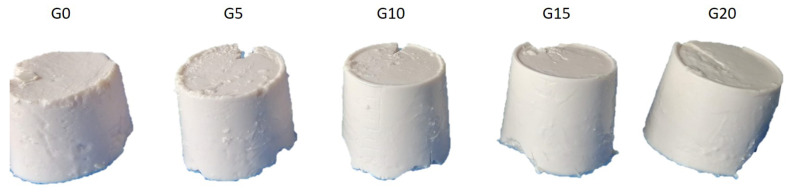
Fermentation-induced PPI gels with 10% protein after 7 days of storage under refrigeration in closed containers. Sample name corresponds to its oil content, e.g., G0: 0% oil, G5: 5% oil, etc.

All PPI emulsions were fermented with Vega^TM^ Harmony and the time required to reach pH 4.5 oscillated between 5.5 and 7 h. All liquid matrices produced firm protein gels during fermentation (Figure 4).

The functionality of the matrices was assessed through their ability to form fermentation-induced protein gels. Protein aggregation occurred at pH values close to the isoelectric point of vicilin (pH 5.5) and legumin (pH 4.8) [32]. Therefore, the obtained gels could be particulate gels composed of randomly aggregated globular protein spheres [33].

**Figure 5:**
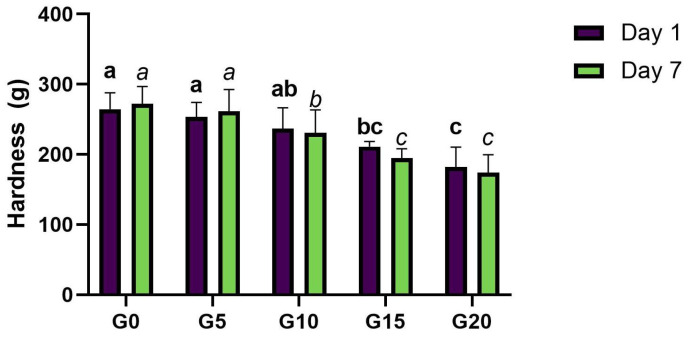
Hardness of fermented PPI gels after 1 and 7 days of storage under refrigeration at 5 °C. Sample name corresponds to its oil content, e.g., G0: 0% oil, G5: 5% oil, etc. Significant differences between samples were highlighted by different letters within day 1 (bold) and day 7 (italics), respectively.

The fermented gels were stored under refrigeration at 5 °C for 7 days, and gel formation was evaluated after 1 and 7 days. Seven days of storage did not have any significant effect on gel hardness in any of the fermented samples (Figure 5). This could be explained by the stability of the gel matrix during storage, including no further activity of the microorganisms which could have affected the microstructure, and also by the storage conditions, since samples were stored in closed containers where drying was avoided. Little syneresis was observed after 7 days in all samples, but it did not affect gel strength according to the results of the texture analysis. Gel strength decreased with increasing olive oil content both in day 1 and day 7 of storage (Figure 5). These results differ from Sim et al., who observed harder pea protein gels with increasing sunflower oil levels [34]. After 7 days of storage under refrigeration, gels with 15% and 20% oil were significantly weaker than all the rest. Moreover, the gel with 10% oil was significantly weaker than G0 and G5 but significantly harder than G15 and G20. These results could suggest a maximum of 10% olive oil in fermented PPI gels, although further experiments are required to confirm that these oil levels would ensure acceptable mouth feeling without compromising on gel hardness.

### 3.6. Oscillation Measurements of Fermented PPI Gels

**Figure 6:**
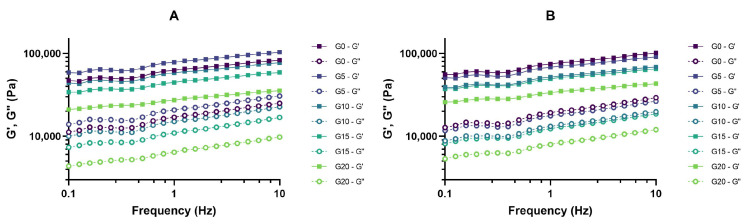
Frequency sweep of fermented PPI gels after (**A**) 1 day and (**B**) 7 days of storage under refrigeration in closed containers. Continuous lines correspond to the elastic modulus (G’) and discontinuous lines to the viscous modulus (G”).

Oscillation tests evaluated the viscoelastic properties of fermented PPI gels. Rheological measurements were taken after 1 and 7 days of storage under refrigeration at 5 °C. All samples presented gel-like behavior, since elastic moduli values (G′) were higher than viscous moduli values (G″) as the frequency sweep in Figure 6 shows.

**Figure 7:**
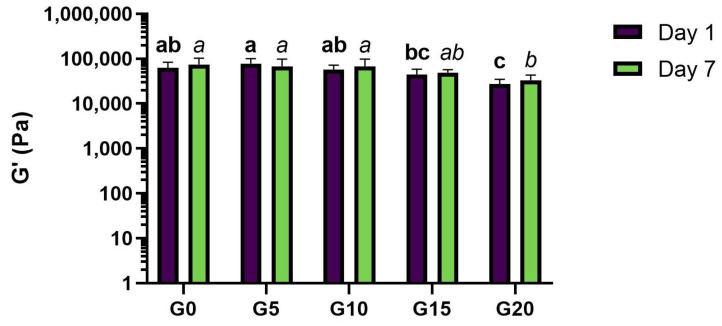
Elastic moduli of fermented PPI gels at 1 Hz after 7 days of storage under refrigeration at 5 °C. Sample name corresponds to its oil content, e.g., G0: 0% oil, G5: 5% oil, etc. Significant differences between samples were highlighted by different letters within day 1 (bold) and day 7 (italics), respectively.

From the frequency sweep data, it can be seen that, independently from the amount of oil added, the obtained gels showed resistance to breakage under the frequency applied. We hypothesize that pea proteins are the main contributors to the elastic modulus of the gel by forming a self-sustained and firm protein network. However, oil content affected rheological properties of the gels. After 7 days of storage, the elastic modulus significantly decreased only with the addition of larger amounts of oil, namely G′ in G20 was significantly different from G′ in G0, G5, and G10. Elastic moduli values at 1 Hz after 7 days of storage can be found in Figure 7, and a similar trend where high levels of oil lead to lower G′ values could be seen after 1 day of storage (data not shown). These results support the outcome of the texture analysis reported in Section 3.5. In this case, 10 or 15% oil could be the critical levels that compromise the elastic modulus. Oil droplets can act as active or inactive fillers in a gel microstructure. Literature defines protein-stabilized oil droplets as active fillers, due to their affinity to the protein matrix [35,36], and they tend to increase the elastic modulus of the gels. However, our results show that the elastic modulus decreased with the highest levels of oil, which suggests that the oil droplets in the studied matrix are not acting as active filler. Inactive fillers, in contrast, have little affinity for the matrix and decrease the elastic modulus of the gels. As Sala explains [37], if the modulus of the filler is lower than the modulus of the matrix, the modulus of the gel system will be affected in the same way no matter if the filler is active or inactive. In this case, we hypothesize that the modulus of olive oil is lower than the modulus of the pea protein structure, which explains a decrease in gel hardness and therefore in the elastic modulus of the gels (directly correlated [38]) with increasing oil levels. However, differences in gel formation could also be attributed to different protein concentration in the water phase even if the protein content was constant in all samples. When keeping protein levels constant and changing oil levels, water content is also affected and varies between samples, meaning that the protein concentration in water is slightly different in each sample.

### 3.7. Effect of Oil on Water-Holding Capacity of Fermented PPI Gels

**Figure 8:**
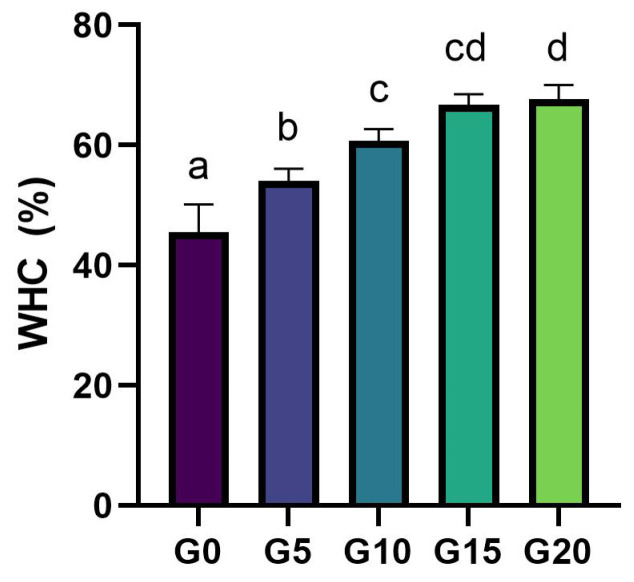
Water-holding capacity (WHC) of fermented PPI gels after 7 days of storage under refrigeration at 5 °C. Sample name corresponds to its oil content, e.g., G0: 0% oil, G5: 5% oil, etc. Significant differences between samples were highlighted by different letters.

The water-holding capacity (WHC) of gels is affected not only by the composition of the gel but also by the gel microstructure, hence by the molecular interactions between matrix components and the structure of the protein aggregates [39]. WHC defines the ability of the matrix to entrap water in the pores of the gel after centrifugation [24]. In this study, WHC was measured after 7 days of storage under refrigeration in closed containers. It significantly increased with increasing olive oil content (Figure 8), and we can already see a difference between gels without oil and gels with low oil levels. This could be explained by an interaction between oil hydrocarbon side-chains and pea protein hydrophobic amino acids, as Wi et al. explained for soy-based meat analogues when liquid oil was added [40]. These interactions could enhance the protein structure and retain more water. Moreover, Wu et al. also observed increased WHC in heat-induced non-animal protein gels and they attributed it to a filling effect of oil droplets [41]. However, our results in Section 3.6 suggested that oil droplets are not acting as active fillers and therefore not interacting with the protein matrix, since the elastic modulus of the gels was decreasing with increasing oil content. Therefore, further work needs to be performed to understand the interactions between olive oil and pea protein matrices to explain the effect of oil on WHC of pea protein gels. WHC results in this study show that the higher the oil content, the smaller the differences in WHC. Additionally, for this aspect, our results suggest 10% oil as a critical olive oil concentration to increase WHC.

### 3.8. Protein Network Visualization in Fermented PPI Gels

**Figure 9:**
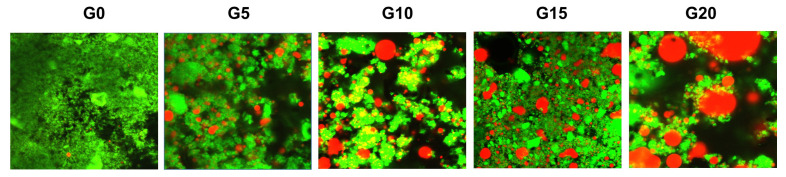
Confocal micrographs of fermented PPI gels after 7 days of storage under refrigeration in closed containers. Sample name corresponds to its oil content, e.g., G0: 0% oil, G5: 5% oil, etc. Proteins are represented in green and oil droplets in red.

Confocal microscopy was used to visualize gel microstructures and the way protein and oil were organized in three-dimensional networks. Representative micrographs in Figure 9 show that all gels were formed by a network of protein aggregates where oil droplets were entrapped. Protein network formation was affected by oil content of the PPI emulsions. With increasing oil content, oil droplets hinder protein-protein interactions and disrupt the protein network, weakening the gels as it was reported in Section 3.5. Moreover, as reflected in Section 3.3, a bi-modal particle size distribution can also be identified in the micrographs, where the oil droplets vary in size in all fermented gels. Gels with higher oil concentrations show larger oil droplets, that could have been formed due to coalescence as explained in Section 3.3.

## 4. Conclusions

Under the conditions applied in the present study, we conclude that PPI is a functional starting raw material for fermentation-induced pea protein gels. The high protein concentration in the PPI and the proper homogenization of PPI emulsions enable protein-protein interactions and the formation of a self-sustained protein network. Protein hydration and homogenization with two pressure stages are two main operations in the production process of a PPI matrix that ensure stability and avoid phase separation over fermentation. This classical procedure was applied in PPI and olive oil matrices to create sufficiently stable emulsions and produce fermentation-induced gels. All emulsions were able to form gels, and their hardness was not significantly affected over storage time under refrigeration at 5 °C in closed containers. A decrease in G′ and an increase in water-holding capacity at higher oil concentrations will result in a softer product, yet less prone to syneresis. Olive oil levels around 10% would be recommended for a PPI matrix with 10% protein content with fermentation-induced gelling properties for plant-based cheese production. Further research performing a deeper characterization of gel microstructure and sensorial analysis to evaluate texture perception will follow. The findings presented in this manuscript should guide plant-based cheese producers in the selection of their raw material and the design of their matrices, and encourage them to develop products with higher quality and improved textural properties.

## Figures and Tables

**Table 1:** ζ-potential of PPI emulsions at t = 0 h and t = 7 h under storage at room temperature. Results are expressed in mV. Sample name corresponds to its oil content, e.g., E0: 0% oil, E5: 5% oil, etc. * Asterisk indicates significant differences between one specific sample at t = 0 h and t = 7 h.

Time (h)	E0	E5	E10	E15	E20
0	−33.4 ± 1.3	−32.4 ± 1.3	−32.7 ± 2.2	−33.3 ± 0.7	−33.1 ± 1.2
7	−36.4 ± 0.8 *	−32.6 ± 0.9	−33.8 ± 1.5	−33.1 ± 1.2	−32.6 ± 1.0

## Data Availability

Not applicable.

## Abbreviations

The following abbreviations are used in this manuscript:

PPI	Pea Protein Isolate
WHC	Water-Holding Capacity
CLSM	Confocal Laser Scan Microscopy
